# The United States Veterinary Medical Association

**Published:** 1887-04

**Authors:** 


THE UNITED STATES VETERINARY MEDICAL ASSOCIATION

Held its semi-annual meeting Tuesday, March 15th, in the amphitheatre of the veterinary department of the University of Pennsylvania.

The comitia minora met at 10 A. M.

The regular session was called at 11 o'clock. The present officers of the association in their chairs: President, A. F. Liautard; vice president, William L. Zuill; secretary, C. B. Michener; treasurer, James L. Robertson.

The report of the comitia minora was first read.

Dr. Michener gave notice that he would offer at the next meeting an alteration of the constitution and by-laws making it imperative that in future all applicants for admission be graduates of recognized colleges.

The following names were presented by the Board of Censors and added to the roll of members: W. H. Martinet, Baltimore; James A. Walrath, New York; Robert C. Jones, Port Jefferson, R. I.; C. Saunders Breed, D. D. Lee, K. Winslow, and E. C. Beckett, Massachusetts; Thomas Bland, Connecticut; George G. Van Mater, Brooklyn; W. E. Cuff, New York; William Rose, Staten Island; T. S. Butler, Ohio, and Dr. Harris, New York.

The library committee failed to report.

Dr. Huidekoper presented, on the part of the committee of intelligence and education, a report from Dr. McInnes, of South Carolina, who stated that the office of veterinarian had been added to the State Board of Agriculture, with a minimum salary, which required twelve tours of the State to be made each year. If more are required, compensation is per diem. There is no veterinarian in the State except the member of the committee.

Dr. Paqnin's report from Missouri stated that more veterinarians were needed there who at the moment of their graduation knew more of anatomy, physiology, and especially pathology of cattle, hogs and sheep. This State had recently established an experimental laboratory.

Dr. W. J. Coates reported from New York the advance made by the passage of a bill regulating veterinary practice, the active interest by some members of the board of health of New York in suppressing contagious diseases which can be communicated to man through the meat and milk, also the increased number of students attending the veterinary colleges.

Dr. Huidekoper reported a diminished number of the lower order of irregular practitioners in Pennsylvania, during the last few years, while the number of qualified veterinarians is increasing, the formation of a State Society within the last two years and the foundation three years ago of the present place of meeting. He reported also the utter lack of laws to protect from contagious diseases, except for contagious pleuro-pneumonia. Of these, glanders and tuberculosis were prevalent throughout the State.

Dr. Hoskins read a report on a uniform standard of examination by the veterinary colleges of the country. The report stated that there is a general desire among the veterinarians and veterinary instructors in different parts of the country in favor of both a uniform and a high standard.

Dr. William L. Zuill, chairman of the committee on contagious diseases, read a report in which he showed that pleuro-pneumonia and tuberculosis prevail in some States to an alarming degree, and that hog cholera and chicken cholera are greatly on the increase.

The meeting then adjourned to an upper room, where a lunch was provided.

In the afternoon session a bill for the benefit of army veterinarians was read by Dr. Pendry, and he was appointed a committee of one to carry it to Washington, and $100 was appropriated to meet his expenses.

Most of the session was occupied by an exhaustive discussion of the subject of pleuro-pneumonia.

Last year a bill was prepared for Congress, which provided for an appropriation of $500,000 to exterminate the disease. At the meeting last fall the association saw the feasibility of changing its form. It was presented, but two medical gentlemen, basing their views on inoculation as relating to the human system, made sworn statements which rendered it necessary to change the form of the bill very materially. Dr. Salmon, Chief of the Animal Bureau at Washington, had prepared everything for the complete annihilation of the disease, but was unable to proceed because of the interference.

Dr. Salmon, Dr. McEachran, president of the Montreal Veterinary Association, McLean, Miller, Winchester and Osgood discussed the subject from every standpoint, and the following resolution was ultimately adopted :

"That the association is convinced that inoculation for contagious pleuro-pneumonia is inapplicable and should not be adopted, and that no animals should be placed in the infected stables until thorough disinfection has taken place ; also, that all animals exposed to or having the disease shall be destroyed."

Action was taken by the association looking to the passage of an act by Congress providing for the proper organization of the Veterinary Commission of the United States Army. This act provides for a Chief, with the rank and pay of Colonel; six Inspecting Veterinarians, with the rank and pay of Captain, and forty assistants, with the rank and pay of Lieutenant.

Dr. D. E. Salmon, of the Bureau of Animal Industry at Washington, spoke of tuberculosis in animals, and said that fully 125,000 to 150,000 human lives were lost every year through the use of milk and meat from tuberculous cattle.

After a continued discussion of the topic, a resolution was adopted calling the attention of the Boards of Health throughout the country to the danger of the disease, and the necessity of rigid and complete inspection of all milk dairies and slaughter-houses, by qualified veterinarians, who should be attached to each Board of Health.

